# K‐means clustering‐based analysis of quantitative ultrafast DCE‐MRI for predicting breast cancer response to neoadjuvant chemotherapy

**DOI:** 10.1002/acm2.70439

**Published:** 2025-12-28

**Authors:** Zhen Ren, Xiaobing Fan, Milica Medved, Frederick M. Howard, Rita Nanda, Hiroyuki Abe, Kirti Kulkarni, Anna Biernacka, Nan Chen, Gregory S. Karczmar

**Affiliations:** ^1^ Department of Radiology The University of Chicago Chicago Illinois USA; ^2^ Section of Hematology and Oncology Department of Medicine The University of Chicago Chicago Illinois USA; ^3^ Department of Pathology The University of Chicago Chicago Illinois USA

**Keywords:** bilateral parenchymal enhancement asymmetry, breast cancer, dynamic contrast enhanced magnetic resonance imaging, k‐means clustering, neoadjuvant chemotherapy

## Abstract

**Purpose:**

Achieving a pathologic complete response (pCR) following neoadjuvant chemotherapy (NAC) is strongly associated with improved survival. This study investigates whether bilateral asymmetry of quantitative perfusion parameters in normal parenchyma from ultrafast dynamic contrast‐enhanced MRI (DCE‐MRI), measured using k‐means clustering (KMC) before NAC, can predict pCR in breast cancer patients.

**Materials and methods:**

Fifty‐six breast cancer patients undergoing NAC with pretreatment ultrafast DCE‐MRI (3–9 s/image at 3T) were enrolled. KMC was used to classify tumor and normal parenchymal voxels into five clusters based on maximum enhancement rate (*A·α*). Ipsilateral‐to‐contralateral (I/C) ratios of background parenchymal enhancement kinetics (kBPE) and tumor kinetics (kT) were compared between pCR and nonpCR groups. Logistic regression models were developed to predict pCR. Statistical tests included bootstrapping, z‐test, chi‐square, and Wilcoxon rank‐sum.

**Results:**

Patients with residual disease showed significantly higher kBPE in the normal‐appearing parenchyma of the ipsilateral breast compared to the contralateral side. Parameters including enhancement rate *α, A·α*, area under the enhancement curve for 30 s *AUC30*, volume transfer constant *K* ^tran^ *^s^*, and rate constant of contrast transfer, *K*_ep_, were significantly higher, while extravascular extracellular space fractional volume, *v*_e_, was significantly lower in the ipsilateral breast parenchyma versus contralateral breast parenchyma for women who have residual disease (*p* < 0.05). A prediction model using kBPE asymmetry alone achieved an area under the curve (AUC) of 0.83. Including tumor kinetics improved the AUC to 0.85.

**Conclusions:**

Bilateral asymmetry of kBPE parameters derived from ultrafast DCE‐MRI using KMC before NAC initiation can predict pCR with high accuracy, providing a **new** minimal‐invasive biomarker for treatment response.

## 1 INTRODUCTION

Despite widespread screening and improvements in treatment, about 30% of patients diagnosed with early breast cancer develop distant metastases.^1^ This is partly because methods of treatment selection have limited efficacy and are largely based on disease extent and receptor subtype.^2^ A treatment that is more precisely tailored to tumor biology and pathophysiology, based on quantitative functional imaging of each patient, could better tailor therapy for each patient and improve outcomes. In addition, imaging markers for response that correlate closely with treatment outcomes can reduce the duration and expense of clinical trials and improve identification of effective drugs by facilitating rapid evaluation.

Many patients with aggressive breast cancer are treated with neoadjuvant chemotherapy (NAC), and pathologic complete response (pCR) after such treatment is an important endpoint that is strongly correlated with improved survival outcomes.^3^ With the introduction of numerous therapies for early‐stage breast cancer, such as carboplatin and pembrolizumab for early triple‐negative breast cancer, more patients are achieving pCR than ever before.^4^, ^5^ However, carboplatin is associated with significant hematologic toxicities including febrile neutropenia, and immunotherapy can result in endocrinopathies such as adrenal insufficiency, which can be permanent and impact quality of life.^6^ Methods that identify patients who would benefit from these agents, and patients who are very likely to achieve optimal responses with less aggressive regimens would have an important impact on patient outcomes.

Dynamic contrast enhanced magnetic resonance imaging (DCE‐MRI) is often used to evaluate breast cancers in patients undergoing NAC and can provide detailed insights into tumor biology and pathophysiology. MRI provides a more accurate assessment of tumor size than other forms of imaging,^7^ and quantitative analysis of MRI can predict the risk of recurrence.^8^ Numerous studies have attempted to use parameters derived from breast DCE‐MRI to predict response to therapy and optimize breast cancer treatment for each patient.^9^, ^10^, ^11^, ^12^, ^13^ Functional tumor volume (FTV), developed by Hylton et al. ^14^ is currently the most established MRI predictive biomarker for response to NAC.^13^ FTV is defined as the metabolically active, perfused tumor volume measured on contrast enhanced MRI.^13^, ^14^, ^15^ Although changes in FTV between preNAC MRI and an early or late cycle of NAC predicted pCR, there was no evidence of a significant association between FTV from preNAC MRI and pCR.^13^, ^16^ Thus, reliable prediction is not yet possible prior to the initiation of NAC. Another study demonstrated the value of adding the tumor apparent diffusion coefficient (ADC) to FTV to produce a more accurate prediction model,^17^ but this only increased the area under the curve (AUC) of receiver operating characteristic (ROC) analysis based on imaging at mid‐NAC and postNAC (pre‐surgery). Recently, Ramtohul et al.^18^ found that the preNAC tumor wash‐in slope assessment from ultrafast DCE‐MRI may predict pCR, but other studies found that tumor wash‐in slope **did not** reliably predict pCR.^19^, ^20^

Measurements of background parenchymal enhancement (BPE) amplitude (i.e. maximum enhancement of parenchyma, referred to in the following as BPE amplitude or ‘aBPE’) from DCE‐MRI have shown promise for early detection of response to therapy.^10^, ^19^, ^21^, ^22^, ^23^ The aBPE may reflect the physiological activity of breast tissue and thus serve as a biomarker for tissue prone to malignant transformation.^24^ A previous study used very low temporal resolution DCE‐MRI to evaluate the aBPE as a marker for response to therapy.^22^ Although decreased aBPE after the first or second cycle of NAC is strongly linked to pCR, a consistent association between pre‐NAC aBPE and pCR has not been convincingly demonstrated.^19^, ^22^ Most previous studies investigating aBPE as a predictor of treatment response measured aBPE in one breast, either the ipsilateral^25^ or the contralateral breast,^11^, ^26^ or averaged aBPE from both breasts.^27^, ^28^ In contrast, the previous work from this laboratory demonstrated that the **difference** between the ipsilateral and contralateral breast (bilateral asymmetry) of parenchymal kinetic parameters (kBPE) from ultrafast DCE‐MRI before the beginning of NAC was significantly associated with pCR.^19^, ^23^ These kinetic parameters are likely reporting on the angiogenic effects of cancers on surrounding parenchyma, and identifying aggressive tumors based on increased angiogenic activity.

Earlier studies indicated that the bilateral asymmetry of kBPE measured from ultrafast DCE‐MRI might reflect angiogenesis, as it results in increased perfusion in the normal tissue of the affected breast compared to the tissue in the contralateral breast.^19^, ^23^ The strong performance of the kBPE bilateral asymmetry as a prognostic marker is consistent with the hypothesis that angiogenic factors secreted by the cancer affect MRI‐measurable parameters in normal parenchyma. The pharmacokinetic parameters we measured from tumors themselves had a lower prognostic value. This suggests that our previous methods did not detect perfusion changes in tumor biology associated with pCR.

In this study, we improved the quantification of the bilateral asymmetry of kBPE parameters measured from preNAC ultrafast DCE‐MRI using the k‐means clustering (KMC) method to enhance the accuracy of bilateral asymmetry of kBPE parameters for predicting pCR. In contrast to the previous studies^19^, ^23^ that compared the bilateral asymmetry of kBPE in the most rapidly enhancing voxels, KMC provides an unsupervised learning method that groups similarly enhancing voxels into clusters for more accurate fitting, so that information from the whole population of parenchymal voxels is used to obtain more stable results.^23^ Furthermore, logistic regression models were used for predicting treatment outcomes. This exploratory study aims to evaluate whether preNAC quantitative MRI parameters derived from KMC analysis can predict pCR in breast cancer patients, providing preliminary evidence of their potential clinical utility.

## 2 MATERIALS AND METHODS

Data from two institutional review board (IRB)‐approved protocols were used in this HIPAA‐compliant study. A retrospective protocol was approved with a waiver of consent. A prospective protocol was approved, requiring written informed consent, which was obtained from all participants.

### 2.1 Patients

Under the retrospective protocol, 51 patients were identified by searching the institutional electronic medical records for the period between December 2016 and March 2023 who met the following criteria: (i) diagnosed with unilateral invasive ductal carcinoma (IDC), (ii) underwent bilateral MRI prior to NAC, (iii) MRI was obtained on a 3T scanner with a 16‐channel breast coil for all exams, including ultrafast DCE‐MRI for 60–80 s after contrast agent administration. In addition, another 12 subjects were recruited prospectively into the study with the same inclusion criteria to increase the sample size. As a result, a total of 63 patients were enrolled in this study. Among them, seven patients were excluded because of low‐quality ultrafast images (*n* = 4), bilateral tumors identified on MRI (*n* = 2), or because they had received endocrine therapy (*n* = 1). Finally, data from 56 patients were analyzed in this study.

Lesions were biopsied prior to NAC to identify receptor status and histologic grade. The breast cancer subtypes included HER2+ (hormone receptor–positive or –negative, HER2+, *n* = 32), triple‐negative (hormone receptor‐negative and HER2‐, *n* = 15) and HER2‐ (hormone receptor positive and HER2‐negative, *n* = 9). Treatment outcome was evaluated based on the residual cancer burden (RCB) index, which was estimated from pathologic sampling of the residual primary breast tumor and regional lymph nodes after completion of NAC. This index is divided into four classes with an increasing amount of residual disease: RCB 0 (pCR), RCB‐I, RCB‐II, and RCB‐III. All clinicopathologic data (age, menopause status, breast cancer subtypes, histologic grade, treatment regimen, pathologic response to NAC, RCB index) were collected through review of medical records.

### 2.2 MRI acquisitions

All patients underwent MRI examination in the prone position on a Philips dStream 3.0 T scanner (Achieva, or Ingenia, Philips Healthcare, Best, the Netherlands) with a 16‐channel bilateral breast coil (MammoTrak or dStream, Philips Healthcare). The scanners are equipped with multichannel RF excitation and RF shimming, resulting in bilateral RF homogeneity. The acquisition protocol included T2‐weighted (T2W) imaging, T1‐weighted (T1W) imaging, diffusion‐weighted imaging (DWI), and conventional and ultrafast DCE‐MRI sequences in the axial orientation. The ultrafast DCE‐MRI protocol consisted of 5 pre‐contrast phases and 8–19 post‐contrast phases. The ultrafast DCE‐MRI were acquired using the following parameters: TR (repetition time) = 2.8–4.8 ms, TE (echo time) = 1.4–2.3 ms, acquisition pixel size = 1.5 × 1.5 mm^2^, slice thickness = 3–4 mm, number of slices = 80–133, field‐of‐view = 320–360 mm, flip angle = 10°–12°, SENSE acceleration factor = 6–8, temporal resolution = 3–9 s. A dose of 0.1 mmol/kg of gadobutrol (Gadavist, Bayer Healthcare Pharmaceutical, Berlin, Germany) or gadobenate dimeglumine (MultiHance, Bracco, NJ) was injected at 2 mL/s, followed by a saline flush of 20 mL at 2 mL/s.

### 2.3 Image segmentation

Data analysis was performed with an in‐house developed MATLAB platform (MathWorks, Natick, MA) and 3D Slicer.^29^ Ultrafast DCE‐MRI images were motion corrected by a nonrigid registration method.^30^ A semiautomatic volumetric segmentation was performed with 3D Slicer using the first pre‐contrast ultrafast DCE‐MRI to obtain ipsilateral and contralateral parenchymal regions of interest (ROIs) which included all breast parenchymal tissue for each patient. The vessel tracking was performed as a part of segmentation. Tumors were manually segmented using postcontrast images by radiologists with 12± years of experience in breast imaging. The vessels and tumors were excluded from the ipsilateral parenchymal ROIs to yield only normal parenchyma before clustering.^19^ Cancerous tumor volume was calculated from the product of the total number of voxels within tumor ROI and the volume of each voxel.

### 2.4 K‐means clustering

K‐means clustering (KMC) is a popular unsupervised clustering technique used for partitioning a dataset into K distinct, nonoverlapping clusters. The goal is to group data points into clusters based on their similarity.^31^

Before clustering, the time course of percent signal enhancement (PSE) was computed for each voxel within the tumor and normal parenchymal ROIs from ultrafast DCE‐MRI data as follows,

(1) $$PSE\;\left(t\right)=\frac{S_{post}\left(t\right)-\bar{S_{pre}}}{\bar{S_{pre}}}\;\times 100\%,$$

where $S_{post}\left(t\right)$ denotes the signal intensity at time ‘t’, and $\bar{S_{pre}}$ is the average signal intensity from the five precontrast images. The PSE(t) was then fitted by an empirical mathematical model (EMM):

(2) $$PSE\;\left(t\right)=A\cdot \frac{{\left(\alpha t\right)}^{2}}{1+{\left(\alpha t\right)}^{2}},$$

where *A* is a scaling constant, and α (min^−1^) is the initial rate of contrast agent uptake. A two‐parameter model is used to avoid over‐fitting the noisy data based on a per‐voxel analysis.

Based on the 3D *A*·α map obtained using the model above, the tumor and normal parenchyma voxels in the ipsi‐ and contra‐lateral breasts were divided into five clusters separately using KMC (*K* = 5). The MATLAB built‐in function ‘imsegkmeans3’ was used to yield reproducible KMC results with clusters sorted in ascending order, by mean value of A·α. *K* = 5 was chosen because *K* = 3 yields clusters with too much variance in A·α, while if *K* > 5, the number of voxels in each cluster may be too small for successful statistical treatment. Therefore, a small number of clusters (*K* = 5) were selected for this pilot study.

### 2.5 Kinetic parameters

Average signal intensity in each cluster was calculated as a function of time and fitted with a three‐parameter EMM as follows:

(3) $$PSE\_c\;\left(t\right)=A\cdot \frac{{\left(\alpha t\right)}^{2}}{1+{\left(\alpha t\right)}^{2}}e^{-\beta t},$$

where *A* and *α* are defined the same way as in Equation 2, and *β* (min^−1^) is the rate of contrast agent washout. The three‐parameter model was used here because with signal averaged by cluster, the signal‐to‐noise ratio (SNR) is high enough to fit the cluster signal enhancement curves more precisely by including *β*. Furthermore, the sum of the individual nonlinear components (Equation 2) does not necessarily produce behavior similar to the original model. The secondary parameter AUC30 for PSE_c(t) was also calculated, which is the integration of Equation 3, i.e.:

(4) $$AUC30=\int_{0}^{t_{30}}A\cdot \frac{{\left(\alpha t\right)}^{2}}{1+{\left(\alpha t\right)}^{2}}e^{-\beta t}\;dt,$$

where $t_{30}$ = 0.5 min.

In addition to the EMM, the extended Tofts model (ETM)^32^ was used to analyze PSE_c(t) to obtain quantitative physiological parameters. Contrast agent concentration curve C(t) in tumor and normal parenchyma was calculated using a previously published method^33^ and fitted with the ETM:

(5) $$C\;\left(t\right)=v_{p}\;C_{p}\left(t\right)+\int_{0}^{t}C_{p}\left(\tau \right)e^{-\frac{K^{trans}}{v_{e}}\cdot \left(t-\tau \right)}d\tau ,$$

where $v_{p}$ is the volume fraction of the plasma space, $K^{trans}$ refers to the transfer constant, $v_{e}$ is the extravascular extracellular space (EES) fractional volume, and $C_{p}\left(t\right)$ is the arterial input function (AIF) taken from the published population AIF model.^34^

For normal parenchymal enhancement, a weighted average from clusters 2, 3, and 4 was calculated for each parameter in two breasts separately by assigning weights 2, 3, and 4, respectively:

(6) $$\begin{array}{cc} kBPE_{ips\_ave} & =\left(2\cdot kBPE_{ips\_c2}+3\cdot kBPE_{ips\_c3}\right.\\ & \;\left.+4\cdot kBPE_{ips\_c4}\right)/9\;, \end{array}$$

(7) $$\begin{array}{cc} kBPE_{con\_ave} & =\left(2\cdot kBPE_{con\_c2}+3\cdot kBPE_{con\_c3}\right.\\ & \;+\left.4\cdot kBPE_{con\_c4}\right)/9, \end{array}$$

where $kBPE_{ips\_ave}$ and $kBPE_{con\_ave}\;$ are the weighted average kBPE parameter in ipsilateral and contralateral normal parenchyma respectively, $kBPE_{ips\_c\#}$ and $kBPE_{con\_c\#}$ refer to the kBPE parameter in the ipsilateral and contralateral parenchyma cluster #, respectively. Clusters 1 and 5 were excluded because they frequently contained outliers due to relatively low parenchymal contrast agent enhancement and noise effects. We used the middle of three clusters in order to obtain more consistent and stable results, especially, given that the ipsilateral/contralateral ratio was used for parenchyma.

To quantify the bilateral asymmetry in the kinetics of normal parenchymal enhancement, the ratio ipsilateral/contralateral (I/C) of the weighted average kBPE parameters (*A*, α, *A*·α, *AUC30*, $K^{trans}$, $v_{e}$ and $v_{p}$) was calculated as follows:

(8) $$kBPE\;I/C=kBPE_{ips_{ave}}\;/\;kBPE_{con\_ave},$$

An I/C ratio > 1 means that a larger kBPE parameter was measured in the ipsilateral normal parenchyma compared to the contralateral parenchyma.

**FIGURE 1:**
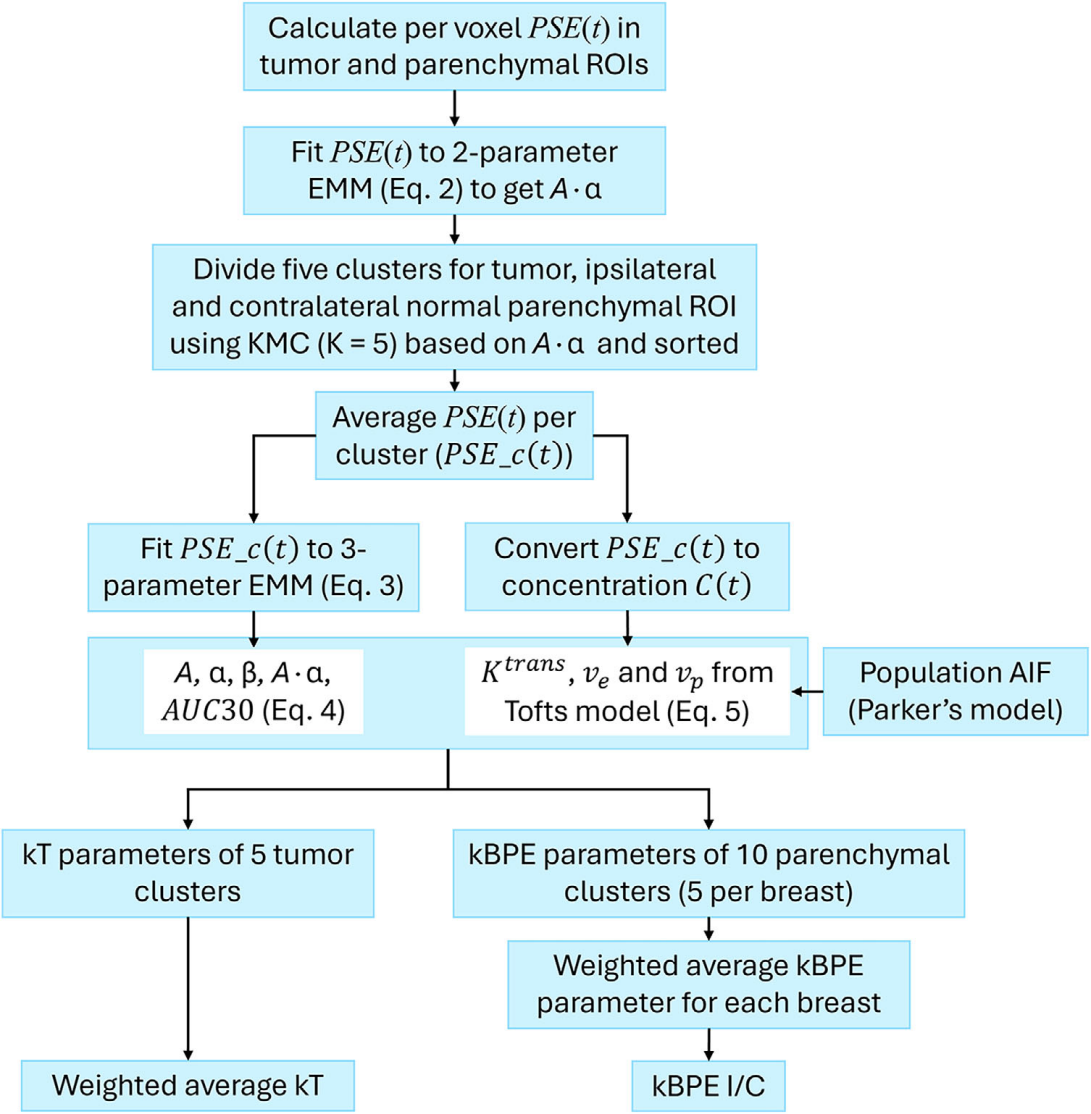
Flowchart of the KMC method‐based image analysis used in this study.

Similarly, weighted average tumor kinetic (kT_ave_) parameters (*A*, α, *A*·α, *AUC30*, $K^{trans}$, $v_{e}$, and $v_{p}$) for cluster 3, 4, and 5 were calculated as follows:

(9) $$kT_{ave}=\left(3\cdot kT_{c3}+4\cdot kT_{c4}+5\cdot kT_{c5}\right)/12,$$

where $kT_{c\#}$ refers to kT parameter in tumor cluster #. Cluster 5 was given the strongest weight because it shows strong enhancement in tumors, the lowest 2 clusters were excluded for tumor because of low SNR. Although simple averages can be used to calculate kinetic parameters between clusters, weighted averages can provide better average parameters by assigning more weight to groups that emphasize cancers due to higher contrast agent uptake. The flowchart of the data analysis is shown in Figure 1.

### 2.6 Statistical analysis

Correlation of the baseline characteristics of the study sample with pathological response to NAC was assessed with a chi‐square test for categorical variables and a Wilcoxon rank sum test for continuous variables. The Wilcoxon rank sum test was also used to compare the weighted average kT_ave_ and the kBPE I/C parameters between patients who achieved pCR and patients with residual diseases (nonpCR). Logistic regression models were generated using combined features: (i) clinical features only, (ii) kT features only, (iii) kBPE I/C features only, (iv) clinical + kT features only, (v) clinical + kBPE I/C features only, (vi) kT + kBPE I/C features only, (vii) and clinical + kT + kBPE I/C features. The candidate clinical features include age, menopausal status, tumor subtype (ER, PR, and HER2 status), and histological grade. To avoid bias in parameter inclusion, we performed an exhaustive search across all candidate parameters and identified the regression model with the highest AUC while restricting the number of parameters to no more than three, in order to minimize overfitting.^35^ The areas under the receiver operating characteristic curves (ROC AUCs) were calculated to evaluate the performance of the logistic regression prediction models. The optimal model was determined as the model with the highest ROC AUC using three or fewer parameters. The bootstrapping method with 1000 bootstrap samples was used for calculating 95% confidence intervals (CIs) of AUC values. Models were compared by calculating the mean of the paired difference in AUC values of bootstrap iterations between models, using the z‐test and testing for nonzero value. Sensitivity and specificity were calculated at the Youden index. A value of *p* < 0.05 was considered statistically significant.

## 3 RESULTS

### 3.1 Patient characteristics

The baseline characteristics of 56 patients are summarized in Table 1. After completion of NAC, 26 patients (46%) achieved pCR and 30 (56%) had residual disease. There is no statistically significant difference (*p* > 0.05) between pCR and nonpCR groups in terms of age, menopause status, receptors status, tumor laterality, baseline histological grade, tumor volume, and treatment regimen (Table 1).

**TABLE 1:** Baseline characteristics of breast patients used in the study.

	Overall (*n* = 56)	NonpCR (*n* = 30)	pCR (*n* = 26)	*p*‐Value
Age (year), median (range)	51 (24–74)	53 (24–74)	50 (30–70)	0.50
Menopause status				0.56
Premenopausal	30 (54)	15 (50)	15 (58)	
Postmenopausal	26 (46)	15 (50)	11 (42)	
ER status				0.86
Positive	33 (59)	18 (60)	15 (58)	
Negative	23 (41)	12 (40)	11 (42)	
PR status				0.33
Positive	21 (38)	13 (43)	8 (31)	
Negative	35(62)	17 (57)	18 (69)	
HER2 status				0.83
Positive	31 (55)	17 (57)	14 (54)	
Negative	25 (45)	13 (43)	12 (46)	
Laterality				0.30
Left	26 (46)	12 (40)	14 (54)	
Right	30 (54)	18 (60)	12 (46)	
Histological grade				0.21
IDC 2	5 (9)	4 (13)	1 (4)	
IDC 3	51 (91)	26 (87)	25 (96)	
Tumor volume (cm^3^) Median (range)	26.0 (0.9–552.4)	19.6 (5.5–552.4)	30.0 (0.9–294.9)	0.34
Treatment				0.83
Anthracycline containing	25 (45)	13 (43)	12 (46)	
Anthracycline free	31 (55)	17 (57)	14 (54)	
Pathologic response				N/A
pCR	26 (46)		26 (100)	
RCB‐I	11 (20)	11 (37)		
RCB‐II	19 (34)	19 (63)		

### 3.2 Prediction of pCR using MRI kinetic parameters

**FIGURE 2:**
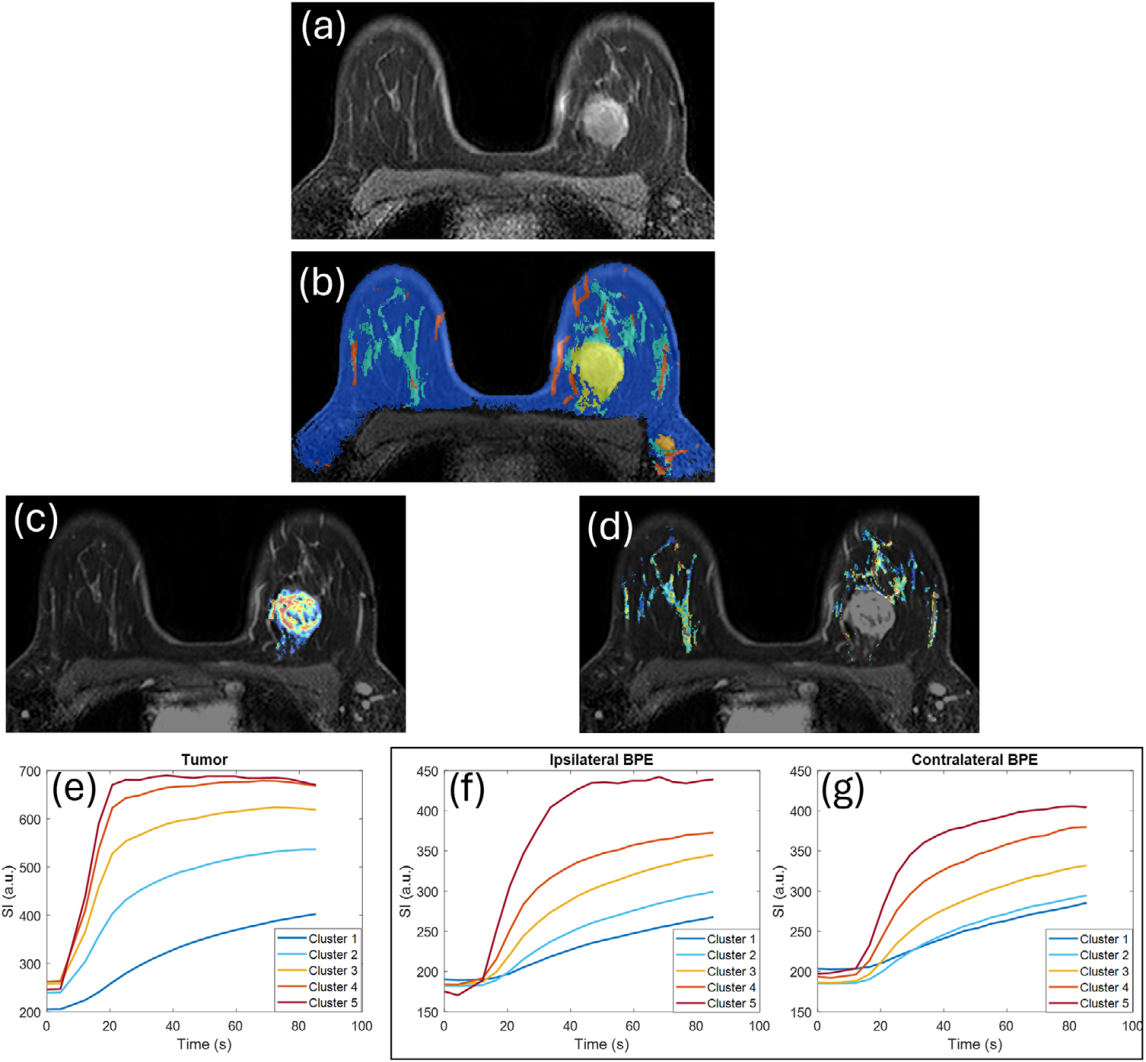
A patient (age = 43 yrs) with IDC in left breast who achieved pathological complete response (pCR) at the completion of neoadjuvant chemotherapy (NAC) (a) image at last time point of ultrafast DCE‐MRI; (b) segmentation, tumor (yellow) was segmented by radiologist, breast (dark blue) and normal parenchymal tissue (light blue) was segmented using 3D Slicer, vessels (red) was tracked and segmented using Hessian filtering method; (c) and (d) five clusters within tumor region of interest (ROI) and normal parenchymal ROI in ipsilateral breast and contralateral breast; (e)–(g) averaged signal intensity curves over time of each k‐means cluster in tumor ROI, ipsilateral and contralateral parenchymal ROIs, respectively.

Figure 2 illustrates the segmentation result for a pCR case, demonstrating the clusters in the tumor ROI and normal parenchymal ROI in two breasts and the signal enhancement averaged from each cluster. As we can see, the average signal curves for cluster 2–4 look similar between the ipsilateral (f) and contralateral (g) breast.

Table 2 presents the weighted averages (± standard deviation) of the parenchymal kinetics bilateral asymmetry parameters (kBPE I/C) of pCR and nonpCR groups, and AUCs with the 95% confidence intervals (CIs) for each parameter for predicting pCR. The nonpCR group had significantly higher *α* (*p* = 0.048)*, A·α* (*p* = 0.001)*, AUC30* (*p* = 0.0001)*, K^trans^* (*p* = 0.00002), and *K_ep_* (*p* = 0.0002) and lower *v_e_* (*p* = 0.01) in the ipsilateral than the contralateral normal parenchyma. The highest AUC for a single feature was 0.83 (95% CI: 0.71–0.93) for parenchymal K^trans^ I/C, followed by *AUC30* I/C and *K_ep_* I/C, the AUC of which was 0.80 (95% CI: 0.67–0.91) for both. Similarly, Table 3 shows the weighted averages (± standard deviation) of tumor kinetic (kT) parameters from preNAC MRI exams, grouped by response to therapy, and AUCs with the 95% CIs of each parameter for predicting pCR. Unfortunately, none of the kT parameters were significantly different between responders (pCR) and nonresponders (nonpCR).

**TABLE 2:** Weighted average (± standard deviation) bilateral asymmetry of parenchymal kinetic parameters by response to NAC.

kBPE I/C	NonpCR (*n* = 30)	pCR (*n* = 26)	*p*‐Value	AUC [95% CIs]
*A*	1.21 ± 0.44	1.19 ± 0.59	0.36	0.57 [0.42, 0.72]
*α*	1.47 ± 0.93	1.02 ± 0.36	0.048	0.66 [0.50, 0.79]
*A·α*	1.66 ± 0.86	1.07 ± 0.26	0.001	0.76 [0.62, 0.88]
*β*	2.06 ± 9.97	5.57 ± 24.53	0.31	0.58 [0.43, 0.74]
*AUC30*	1.79 ± 1.29	1.05 ± 0.31	0.0001	0.80 [0.67, 0.91]
*K^trans^*	1.45 ± 0.56	1.03 ± 0.12	0.00002	0.83 [0.71, 0.93]
*v_e_*	0.88 ± 0.79	1.05 ± 0.24	0.01	0.70 [0.55, 0.83]
*K_ep_*	5.00 ± 8.13	1.06 ± 0.42	0.0002	0.80 [0.67, 0.91]
*v_p_*	3.08 ± 3.88	1.44 ± 0.85	0.26	0.60 [0.45, 0.76]

**TABLE 3:** Weighted average (± standard deviation) MRI tumor kinetic parameters by response to NAC.

Tumor parameters	NonpCR (*n* = 30)	pCR (*n* = 26)	*p*‐value	AUC [95% CIs]
*A*	1.49 ± 0.44	1.52 ± 0.43	0.63	0.54 [0.39, 0.71]
*α* (min^−1^)	13.7 ± 7.84	12.0 ± 7.6	0.27	0.59 [0.43, 0.74]
*A·α* (min^−1^)	21.1 ± 13.4	19.5 ± 14.3	0.45	0.56 [0.40, 0.72]
*β* (min^−1^)	−0.04 ± 0.16	−0.05 ± 0.13	0.86	0.51 [0.36, 0.66]
*AUC30*	0.56 ± 0.20	0.55 ± 0.20	0.98	0.50 [0.33, 0.66]
*K^trans^* (min^−1^)	0.21 ± 0.11	0.18 ± 0.09	0.49	0.56 [0.41, 0.71]
*v_e_*	0.33 ± 0.17	0.33 ± 0.19	0.65	0.54 [0.37, 0.69]
*K_ep_* (min^−1^)	0.74 ± 0.32	0.67 ± 0.26	0.31	0.58 [0.43, 0.73]
*v_p_*	0.0062 ± 0.0067	0.0060 ± 0.0059	0.90	0.55 [0.40, 0.71]

The reverse constant *K_ep_* = *K^trans^/v_e_*.

**FIGURE 3:**
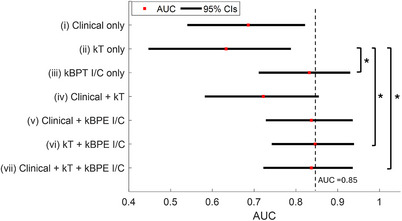
ROC AUCs and the 95% CIs of the prediction models listed in Table 4. The dashed line refers to AUC = 0.85. The asterisks denote statistically significant differences at the *p* < 0.05 level.

Finally, Figure 3 shows the estimated AUC values and the 95% CIs for logistic regression models generated with combined features, which are listed in Table 4. Including kBPE I/C features results in higher AUC compared to the models using clinical features or kT features alone. Models with kBPE I/C features alone or where kBPE I/C features were added to kT or kT and clinical features had significantly higher AUC than the model using kT features alone. The highest AUC (= 0.85; 95% CI: 0.74–0.94) is achieved for the model that combined kT and kBPE I/C features. The model that combined one from each clinical, kT and kBPE features had a slightly lower AUC (= 0.84; 95% CI: 0.72–0.94).

**TABLE 4:** Summarizes the diagnostic performance of the logistic regression prediction models with the inclusion of features and the diagnostic performance.

Prediction models	Features	AUC [95% CIs]	Sensitivity	Specificity	Accuracy
(i) Clinical only	Age, PR, HER2	0.69 [0.54, 0.83]	93.3%	38.5%	0.64
(ii) kT only	*A, v_e_*, *K_ep_*	0.63 [0.45, 0.79]	80.0%	53.8%	0.54
(iii) kBPE I/C only	*A, K^trans^*	0.83 [0.71, 0.93]	73.3%	88.5%	0.68
(iv) Clinical + kT	PR, HER2, kT (*α*)	0.72 [0.58, 0.86]	70.0%	76.9%	0.66
(v) Clinical +kBPE I/C	HER2, kBPE (*AUC30, K_ep_*)	0.84 [0.73, 0.94]	80.0%	76.9%	0.77
(vi) kT + kBPE I/C	kT(*A*), kBPE (*v_e_*, *K_ep_*)	0.85 [0.74, 0.94]	63.3%	88.5%	0.77
(vii) Clinical + kT + kBPE I/C	ER, kT (*K_ep_*), kBPE (*K^trans^*)	0.84 [0.72, 0.94]	80.0%	80.8%	0.68

## 4 DISCUSSION

Our study demonstrated that the bilateral asymmetry of kBPE parameters measured from preNAC ultrafast DCE‐MRI can discriminate between pCR patients and patients with residual disease post‐treatment more effectively than analysis that only takes tumor kinetics and/or clinical features into account. The majority of the MRI‐based kBPE I/C features were significantly associated with pCR with the highest AUC (= 0.83) found in *K^trans^* I/C. This performance is better (higher AUC values) than using the clinical features or tumor kinetics alone, though not all differences were statistically significant with our sample size. Calculating kBPE I/C ratio as a prognostic indicator is more effective than using aBPE from a single breast or its average in the two breasts as these measurements can vary significantly between individuals and are strongly associated with hormonal *milieu* and cardiac function. Comparison of perfusion in the ipsilateral to the contralateral breast reduces the effects of interpatient variations in the arterial input function and cardiac function.^23^, ^36^

In earlier studies, various prediction models have been developed to assess NAC outcomes early, with most based on clinical features,^37^, ^38^, ^39^ MRI tumor kinetic features,^13^, ^16^, ^17^, ^40^ a combination of the two^18^, ^41^ or tumor texture^42^ only, and information from mid or postNAC was needed to achieve a high AUC. Ramtohul et al. ^18^ reported a model based on 50 patients that combined preNAC wash‐in slope in tumor, HER2 status, and tumor‐infiltrating lymphocytes for identifying pCR and RCB‐I from RCB‐II. Of the MRI features analyzed in Ramtohul's study, only the wash‐in slope in tumor was associated with pCR with an AUC of 0.76. In contrast, two other studies assessing preNAC tumor kinetic parameters found no significant association with pCR.^19^, ^20^

To quantify the bilateral asymmetry in normal parenchymal enhancement, our earlier studies^19^, ^23^ used the most rapidly enhancing 10% or 20% of parenchymal voxels to calculate kBPE parameter asymmetry, under the hypothesis that the most rapidly enhancing voxels are the best indicators of changes in perfusion of normal tissue due to angiogenic factors produced by cancers. However, the selection of a fixed threshold is arbitrary, and the majority of voxels with adequate SNR are not utilized in this approach. In the current study, we sought to maximize the number of voxels used in the analysis by applying the KMC method, which provides a robust and reproducible approach to classifying and comparing regions with different perfusion characteristics. By clustering voxels with similar uptake curves together, it is possible to obtain meaningful estimates of kinetic parameters. Conversely, averaging all voxels‐of‐interest into a single enhancement curve can cause distortion and introduce bias in the estimated parameters. Because of the non‐linearity of the EMM, the model parameters fitting the average curve would differ from the average of the model parameters fitting individual cluster curves. In addition, the clustered ROI‐based analysis is less influenced by noise and easier to implement compared to the method introduced previously.^23^ Thus, the KMC approach allows us to more meaningfully quantify the bilateral asymmetry of kBPE parameters.^31^

Earlier studies indicated that the bilateral asymmetry of kBPE measured from ultrafast DCE‐MRI might reflect angiogenesis, as it results in increased perfusion in the normal tissue of the affected breast compared to the tissue in the contralateral breast.^19^, ^23^ The high diagnostic performance of the kBPE bilateral asymmetry prognostic markers is consistent with the hypothesis that angiogenic factors secreted by the cancer affect MRI‐measurable parameters in normal parenchyma. The pharmacokinetic parameters we measured from tumors themselves had a lower prognostic value. This suggests that our current methods do not detect perfusion changes in tumor biology associated with pCR. In future work, we will explore additional approaches such as incorporating FTV and further increasing temporal resolution for tumor MRI acquisition and analysis.^33^ These efforts aim to identify parameters that are more sensitive to tumor aggressiveness and angiogenic activity. Because angiogenic activity and perfusion in cancers can occur at very high rates, detection and characterization may require even higher temporal resolution than our current ultrafast protocol provides. We also plan to validate our method in larger, multicenter cohorts and to develop more comprehensive prediction models that integrate established clinical and imaging predictors with our proposed biomarkers.

This study has several limitations. **1)** The relatively small, single‐center sample size limited the number of parameters that could be included in the logistic regression models and precluded external validation. In addition, potential variability in ultrafast DCE‐MRI implementation across scanners and vendors may affect generalizability. As multicenter data become available, more complex models can be developed and externally validated to improve performance. **2)** The temporal resolution of the study samples ranged from –9 s per image, which introduced some variability in the dataset. Ideally, all cases should have the same temporal resolution. High temporal resolution is required to accurately measure many of the kinetic features that have prognostic value, and temporal resolution of better than 3 s may be more effective. **3)** The accuracy of v_e_ estimation may have been reduced by the fact that the contrast washout was only followed for 60 s. **4)** A population AIF was used for pharmacokinetic calculations rather than a patient‐specific AIF. The population AIF may not accurately reflect the individual variations in blood flow and may potentially lead to inaccuracies in *K^trans^* calculations. However, in this study, the most effective prognostic parameters came from comparisons of the ipsilateral and contralateral breasts. This comparison reduces, but does not eliminate, effects of interpatient variation of the AIFs. In future studies, we would address these limitations to increase prognostic accuracy. **5)** Two different contrast agents, MultiHance and Gadavist, were used in this study. Ideally, the same contrast agent should be used to avoid effects on the calculated parameters. The Tofts model involves contrast agent concentration which is affected by the relaxivity of the contrast agent. However, the relaxivity of the contrast agent would affect both AIF and tissue proportionally. Therefore, the different relaxivities of MultiHance and Gadavist would have minimal effects on the parameters used in this study. Furthermore, the ipsilateral/contralateral ratio would eliminate more effects of the different contrast agent relaxivities on the parameters. **6)** The B1 field inhomogeneity was not corrected for the parameters calculated in this study. In future, incorporating B1 mapping and correction would be needed to increase the accuracy of kBPE asymmetry findings.^43^ **7)** Finally, the number of clusters and weights of average used in this study may not be optimal. It is critical to choose the appropriate number of clusters in order to obtain quality and interpretability of results. Various weighting should be evaluated to enhance the results. In future, more sophisticated methods should be used to determine optimal number of clusters and weights of average.

In conclusion, we introduce an effective new method for predicting the response of breast cancer to neoadjuvant chemotherapy before the beginning of therapy, based on quantitative analysis of ultrafast DCE‐MRI. We demonstrate that quantifying bilateral asymmetry in parenchymal kinetic parameters using k‐means clustering provides independent predictive markers for NAC outcome. Increased kBPE parameter asymmetry in more aggressive cancers is likely due to the angiogenic effects of cancers on surrounding tissue. In the future, we will investigate definitive predictive models with larger, independent cohorts. We would also test the combination of these new prognostic markers with more established markers such as functional tumor volume and the amplitude of background parenchymal enhancement.

## AUTHOR CONTRIBUTIONS STATEMENT

Zhen Ren contributed to the conception, design, data collection, software and methodology, visualization, analysis, drafting, and critical review. Xiaobing Fan contributed to the design, software and methodology, analysis, and critical review. Milica Medved contributed to the analysis and critical review. Frederick M. Howard and Rita Nanda contributed to funding acquisition, data collection, and review. Hiroyuki Abe contributed to funding acquisition, analysis, and review. Kirti Kulkarni contributed to analysis and review. Anna Biernacka and Nan Chen contributed to review. Gregory S. Karczmar contributed to supervision, funding acquisition, and review.

## CONFLICT OF INTEREST STATEMENT

The authors declare no conflict of interest.
